# Scabies in Resource-Poor Communities in Nasarawa State, Nigeria: Epidemiology, Clinical Features and Factors Associated with Infestation

**DOI:** 10.3390/tropicalmed3020059

**Published:** 2018-06-04

**Authors:** Uade Samuel Ugbomoiko, Samuel Adeola Oyedeji, Olarewaju Abdulkareem Babamale, Jorg Heukelbach

**Affiliations:** 1 Parasitology Unit, Department of Zoology, University of Ilorin, PMB 1515 Ilorin, Nigeria; oyedejisamed@gmail.com (S.A.O.); olas4nice2004@yahoo.co.uk (O.A.B.); 2 Department of Community Health, School of Medicine, Federal University of Ceará, Fortaleza CE 60430-140, Brazil; heukelbach@web.de; 3 College of Public Health, Medical and Veterinary Sciences, Division of Tropical Health and Medicine, James Cook University, Townsville 4811, Australia

**Keywords:** scabies, epidemiology, parasitic skin disease, cross-sectional study, Nigeria

## Abstract

Epidemiology and clinical features of scabies remain largely unknown in Nigeria’s rural communities. To fill this gap, we performed a cross-sectional study in three rural communities in north central Nigeria. A total of 500 individuals were included and examined for scabies infestation; a questionnaire was applied to collect socio-demographic and behavioral data. Scabies was diagnosed in 325 (65.0%) participants. Excoriations (68.6%), vesicles (61.8%), and papules (58.8%) were common skin lesions. Itching was the most common symptom (77.5%); 64% complained of sleep disturbances. Lymphadenopathy was identified in 48.3%. Lesions were most commonly encountered on the abdomen (35.5%), inguinal area (19.1%), and interdigital spaces (14.2%). Poverty-related variables, such as illiteracy (OR: 7.15; 95% CI: 3.71–13.95), low household income (7.25; 1.19–88.59), absence of a solid floor inside house (12.17; 2.83–52.34), and overcrowding (1.98; 1.08–2.81) were significantly associated with infestation. Individual behavior, such as sharing of beds/pillows (2.11; 1.42–3.14) and sharing of clothes (2.51; 1.57–3.99), was also highly significantly associated with scabies. Regular bathing habits (0.37; 0.24–0.56) and regular use of bathing soap (0.36; 0.21–0.53) were protective factors. Scabies is extremely common in the communities under study and is associated with considerable morbidity. The disease is intrinsically linked with extreme poverty.

## 1. Introduction

Scabies is a common contagious parasitic skin disease and a public health problem, mainly in tropical and subtropical countries [1,2]. Hundreds of millions of people suffer from infestation in impoverished urban and rural communities worldwide [3,4,5,6]. Outbreaks of scabies in closed groups have been reported particularly from high income countries, but the disease is more common in resource-poor communities in low and middle income countries in tropical climate zones [7,8,9]. High prevalence and re-infestations in endemic settings are correlated with armed conflicts, homelessness, crowding, and communal use of clothes, beds, and pillows [10,11]. Between 18% and 70% of people are reported to be affected in resource-limited communities in India, on south Pacific islands, and in Australian Aboriginal communities [1,8,12], with severe morbidity being common, such as abscess formation, lymphadenopathy, and post-streptococcal glomerulonephritis [8,13,14,15]. Control and prevention strategies by chemotherapy require considerable public health services and home resources, since treatment is often cumbersome and stressful [2,16].

The prevalence of scabies disease remains largely unknown in Nigeria, in the face of many other health problems considered of more severe morbidity [17,18,19]. Only a limited number of hospital-based studies have described scabies and other skin diseases [20,21,22,23,24]. However, the upsurge in communal clashes and terrorist insurgence have increased the number of refugees and infectious diseases in all cardinal zones of the country, which poses vulnerable communities at higher risk for scabies infestation. To provide information on the epidemiology of scabies in Nigeria, a cross-sectional community-based study of scabies infestation was conducted in impoverished rural communities in north central Nigeria.

## 2. Materials and Methods

### 2.1. Study Area

The study was conducted in three indigenous communities in the Lafia Local Government Area (LGA) of Nasarawa State (north central Nigeria). State health workers had communicated endemicity of scabies from these communities. The areas (latitude 8°32′ N; longitude 8°18′ E) are largely settlements with an estimated population of 3300 inhabitants (National Population Commission, 2007). The climate condition in the state is tropical with a mean daily temperature of 40 °C and relative humidity of 90% during the dry season (November–April).

In the area there have been several violent communal disturbances, with consequently many families being displaced and living under inappropriate housing conditions. Most houses are built with clay or palm products, and there is no electricity, nor a structured community waste disposal system. The inhabitants are predominantly Muslims. The people are largely illiterate subsistence farmers of food crops and cattle herders, with 84% of the households on a monthly per capital income of <N18,000 (equivalent to US$50 at the time of study), the national minimum wage. Human and animal waste is littered around compounds and is widely dispersed during heavy rains. Rivers and streams serve as the major source of water supply. Roads are caliche-topped. Public schools and health centers in the areas are deplorable and poorly staffed. School-aged children (locally called ‘Almagiri’) are compelled to solicit for food and finance for their daily existence.

### 2.2. Study Design

For the purpose of this study, the three communities under study were considered as one study area. Prior to the study, a complete household census conducted in the study area identified 390 households (about 3300 individuals), which were listed and subsequently numbered. Using the Epi Info Software package (version 6.04d), we estimated that a population of 344 individuals was sufficient for the prevalence study with 80% power and 95% confidence level. Considering a safety margin (due to expected high non-participation) and a cluster effect (household sampling), a total of 209 households comprising 1050 individuals were selected using a random number generator (Epi Info version 6.04d, Centers for Disease Control and Prevention, Atlanta, GA, USA).

The study was conducted during the dry season (data collection November 2016–April 2017). All members in the selected households were eligible for the study except for individuals who declined participation and those who had not spent at least five days per week in the previous two months in the study area. Those who consented were registered and interviewed to obtain information on their biodata (name, sex, age, and educational background), socioeconomic information, and environmental and behavioral variables, using pre-tested structured questionnaires.

Thereafter, the entire skin surface including the genital areas of each participant was carefully examined clinically for scabies infestation. Children aged <10 years were examined in the presence of their parents or caregivers. In the absence of any household members, the houses were revisited three times.

### 2.3. Case Definition and Skin Examination

Within the realm of this study, scabies was defined on the basis of a symptomatic description proposed and used by our group previously [9,10,13]—namely, presence of at least two of the following requirements: 1. one or more typical lesions for longer than 2 weeks; 2. pruritus that intensified at night; 3. at least one more family member with similar lesions. Typical skin lesions included papules, vesicular rash, and nodules.

For description of topographical distribution of lesions and parts of the affected skin surfaces, the entire body surface was vertically divided into left and right sides. Each side was subdivided into interdigital spaces, hand, wrist, arm, elbow, axilla, leg, foot, abdomen, thorax, mamilla/perimammillar area, back, buttock, genital/inguinal area, and head (scalp/neck/face) [6]. Primary lesions were distinguished as macular, papules, crusted papules (if a tiny hemorrhage crusts), vesicles, and nodules [3,6,8,25]. Excoriations on skin and bacterial superinfection were noted when pustules, abscess, or suppuration were observed. Lymph nodes were palpated to confirm lymphadenopathy while the intensity of itching was assessed semi-quantitatively and graded as absent, light, moderate, and heavy, using visual ordinal scales. To guarantee privacy of participants, all individuals were examined in a well-lighted room provided in the households. Examinations were performed by one male investigator (SAO) extensively trained in diagnosis of scabies, and assisted by a female senior nursing officer of the Dermatology unit of Nasarawa Central Hospital.

### 2.4. Statistical Analysis

Data were double-entered into a database and crosschecked for entry errors, using SPSS version 16.0 for Windows (SPSS Inc., Chicago, IL, USA). Chi-squared statistics was applied to determine the significance of differences of relative frequencies between groups. Age groups were defined as follows: ≤10 years, 11–15 years, 16–20 years, ≥21 years. Bivariate analysis and multivariate logistic regression models were applied to identify independently associated variables measured on the prevalence of scabies in the communities. Variables were checked for collinearity before inclusion in the multivariate model.

### 2.5. Ethical Considerations

The study protocol for this study was approval by the Ethical Review Committee of the University of Ilorin, Nigeria and the Primary Health Care Unit of the Lafia Local Government Area of Nasarawa State. Prior to the study, informed consent was obtained from adult participants and from the parents or legal guardians of minors after detailed explanation of the study protocol. In accordance with the ethical review committee requirements, patient information was made confidential.

## 3. Results

### 3.1. Characteristics of Study Population

The target population consisted of 1050 individuals (546 males and 504 females) belonging to 144 households. Seventy-eight individuals failed inclusion criteria, and 38 moved to neighbouring communities during the study. Of the remaining 934 individuals, the data records of 65 were incomplete (interview data or clinical examination). A total of 369 females declined participation for religious and cultural reasons.

Consequently, the study population consisted of 500 individuals, comprising more males (*n* = 429) than females (*n* = 71). The age of individuals ranged from 1 to 34 years (median = 14 years), and the household size ranged from 2 to 14 persons (median = 6).

The characteristics of the study population are presented in Table 1. The study population was primarily illiterate, with an illiteracy rate of almost 90%. Males and age group >21 years were disproportionately highly represented in the study population.

### 3.2. Scabies Prevalence and Clinical Features

A total of 325 (65.0%) participants were diagnosed with scabies. Excoriations, vesicles, and papules were the most common skin lesions (Table 1). There were no cases of crusted scabies. The prevalence of scabies stratified by socio-demographic and cultural factors is presented in Table 2. Age groups <15 years showed highest prevalences (accounting for 76.3% of all infected cases), and infestation was significantly more common in females (74.6%) than in males (60.6%).

Signs and symptoms associated with scabies are presented in Table 3. Itching was the most common symptom (77.5%), with 56% presenting severe itching; 52% complained of itching-related sleep disturbance. Lymphadenopathy was identified in about half of the infected cases, commonly in the inguinal and cervical regions.

The topographic distribution of lesions is shown in Table 3. Lesions were most commonly encountered on the abdomen (35.4%), inguinal area (19.1%), and interdigital spaces (14.5%).

### 3.3. Factors Associated with Infestation

Table 2 presents the bivariate analysis of factors associated with scabies infestation. Prevalence in <16-year-olds was significantly higher than in older age groups. Poverty-related variables such as illiteracy, low household income, inadequate housing, unemployment, and overcrowding were significantly associated with scabies. Sharing of beds/pillows and sharing of clothes were also highly significantly associated with infestation. Regular bathing habits and regular use of bathing soap were protective factors. In multivariate logistic regression analysis, poverty-related variables remained independent factors significantly associated with infestation (Table 4).

## 4. Discussion

Our study represents the first systematic community-based study on scabies conducted in Nigeria, revealing an extremely high prevalence and morbidity of scabies. The disease was associated with poverty-related variables even within the communities under study, which can be characterized as extremely resource-poor, with precarious living conditions. The high prevalence recorded in the present study indicates the under-recognition of the disease in resource-poor communities, and difficult access to the health system. The prevalence of 65% is comparable to studies in specific and high-risk populations worldwide. For example, prevalences were similar or even higher in a Bangladeshi Islamic religious school (61%) [26], displacement camps in Sierra Leone 67%) [27], Thailand orphanages (87%) [28], a Korean leprosarium (87%) [29], and in a rural village in Papua New Guinea (80%) [30]. Other studies from Nigeria reported lower prevalences of 5% to 57% [20,21,22]. Other African countries (Cameroon—18% [31], Malawi—36% [32]), as well as Cambodia (4.3%) [33], Brazil (9–10%) [9,10,34], and Fiji (24%) [2] also reported lower prevalences, as compared to our study.

The endemicity of scabies and the associated burden have been attributed to a wide range of intervening factors previously, including socio-economic factors, overcrowding, and behavioral and environmental variables [1,10,13,35]. Consistent with these reports, our findings confirmed poor housing conditions and behavior such as sharing of beddings and pillows—which may serve as fomites—as important risk factors. Overcrowding in the study area was worsened by internal migration of refugees from neighboring communities due to recent communal clashes and terrorist insurgencies. In fact, crowding is a known risk factor for scabies, and has been reported previously in several studies from endemic areas in Egypt [36], Sierra Leone [27], Mali [33], Brazil [10], India [26], and Thailand [28]. Similarly, other proxies for poverty such as unemployment, low income, communal use of clothing, and illiteracy were significantly associated. Multivariate analysis indicated the importance of hygiene habits as independent protective factors.

Another major outcome of this study is the uneven distribution of scabies and its morbidity. Our data show that scabies in the female study population was significantly higher, as compared to males. However, considering the high non-participation rate in females, this figure must be taken with care. There was also a considerable variation in prevalence with age. Children of school age were more frequently infested than the older age groups. This is in agreement with other studies from endemic areas [9,37], indicative of the high frequency of interaction and poor hygiene that enhances transmission amongst these highly mobile age groups. In our study area, these age groups are mostly schoolchildren of private Islamic schools that are sent out by school owners to roam around the streets as so-called Almajiri (beggars) and beg for food and money (OAB, personal observation). The observed occurrence of scabies in the middle and older age groups >21 years may be attributed to sustained contact with infested children, especially in the female population.

We also observed that the topographical distribution of the morbidity-associated features of scabies varied in the population. More than 14% of the infested had more than one type of lesion in various topographic sites, commonly on the abdomen, inguinal/thigh, interdigital space, hands, and wrists, confirming previous reports [3,6,9]. In our study area, a climate-determined behavior in which people, particularly male children, expose greater parts of the body and maintain prolonged close physical contact facilitates transmission of scabies mite. This partly explained why lesions commonly occurred on the abdomen, hands, and wrists in this study.

Our data further show that the prevalence of both itching and excoriation in the affected population was high (77.5% and 68.3%, respectively). Scabies-related itching is a host allergic immune response to mite products [38]. Usually, in resource-limited settings, secondary bacterial infection is common due to poor hygiene conditions and overcrowding [39]. Intense itching and scratching result in skin breaks and facilitates secondary bacterial infection among the affected population [9,13,32,37,40]. Lymphadenopathy has also been well correlated to secondarily-infected scabies lesions in individuals [9,34]. In the current study, high proportions of lymphadenopathy and itching were reported in the affected population. Similar conditions are, however, not uncommon with other parasitic skin disease, such as tungiasis and cutaneous larva migrans [17,41]. Severe itching has also been reported to induce sleep disturbances in scabies-affected individuals. Although other diseases may induce sleep disturbance, the observation that sleep was more often disturbed at a time that coincides with the peak activities of the sarcoptic mites indicates the involvement of this mite. Sixty-four percent of the population with scabies reported sleep disturbance. This is comparable to 77% of cases reported previously from Brazilian communities [6].

Scabies is increasingly recognised as a common parasitic skin disease in Nigerian children [20,21,22], particularly in poor rural communities where important infrastructural facilities including health care services are unavailable or inadequate. With the current socioeconomic and lifestyle patterns of people where living conditions are precarious—e.g., families sharing clothing and bed space—scabies will be continuously endemic, with high prevalences.

The cost of and access to healthcare services is prohibitive and difficult for many individuals at risk living in the study area and elsewhere in Nigeria in similar settings. Thus, traditional medication, which often complicates disease conditions, is an alternative choice of care by many affected people. Alleviating the scourge of potentially preventable and treatable diseases such as scabies is fundamental in public health service. In Nigeria, and indeed many other African countries, identification and treatment of cases appears to be the only management option in the face of paucity of reliable epidemiological data for control programs. The effectiveness of oral ivermectin in the treatment of scabies and other parasitic diseases such as pediculosis, lymphatic filariasis, onchocerciasis, and intestinal helminthiasis has been widely reported from endemic areas in Africa, South America, and Pacific Islands [1,3,8,13,39]. In endemic communities, control programs by oral ivermectin chemotherapy could be integrated into other existing parasite control programs with strong advocacy on health education, training of health personnel, and surveillance.

Our study is subject to several limitations, and internal and external validity of data may have suffered from the extremely difficult field conditions under which this study took place. Given the considerable difference in participation rates between males and females, and under-representation of adults, prevalence data may have suffered from participation bias. Many female non-participants declined due to cultural and religious beliefs. During field work in the community, we sought to discern other reasons for the striking gender-driven non-participation, but could confirm repeatedly during interviews with community members that the major reasons for non-participation in females in fact were of socio-cultural origin, and not related to symptomatic scabies infestation status (SAO, personal communication). Diagnosis of scabies was based on clinical features; microscopic examination of skin scrapings, videodermatoscopy, and bacteriological testing to validate the presence of mite and bacterial infection in lesions, could not be conducted in this extremely difficult field setting. In some cases diagnosis may have been inaccurate; for example, untreated onchodermatitis (due to onchocerciasis) may have been misclassified as scabies. We aimed to reduce possible diagnostic error by systematic training and supervision of field investigators, and by meticulously adapting to the diagnostic approach as proposed by Heukelbach & Feldmeier [13]. Despite the limitations mentioned, we believe that this population-based study from typical impoverished communities in Nigeria may reflect the situation in similar communities throughout the country.

## 5. Conclusions

Scabies is extremely common in the rural Nigerian communities under study, and associated with considerable morbidity. We have confirmed that even in the least developed and precarious communities, poverty-related variables are important risk factors for infestation and that hygiene habits may still have a protective effect, even in settings with extremely high transmission pressure. Communal clashes and disturbances related to displacement, overcrowding, and unemployment may further increase prevalence and scabies-related morbidity. Given the risk of sequelae related to chronic infestation and bacterial superinfection, an urgent response from the health care sector is mandatory. Intervention measures may be integrated into existing helminth control programs based on oral ivermectin mass treatment.

## Figures and Tables

**Table 1:** Characteristics of study population (*n* = 500).

Variable	N (%)
Sex	
Male	429 (85.8)
Female	71 (14.2)
Age group (years)	
≤10	104 (20.8)
11–15	232 (46.4)
16–20	117 (23.4)
≥21	47 (9.4)
Community	
Lafia Municipal	175 (35.0)
Lafia East	160 (32.0)
Lafia North	165 (33.0)
Education	
Illiterate	440 (88.0)
Primary	48 (9.6)
Post primary	12 (2.4)
Presence of scabies-typical lesions	
Yes	325 (65.0)
No	175 (35.0)
Type of lesions	
Papules	191 (58.8)
Crusted papules	105 (32.3)
Vesicles	201 (61.8)
Macules	160 (49.2)
Pustules	132 (40.6)
Excoriations	223 (68.6)

**Table 2:** Prevalence of scabies and bivariate analysis of socio-demographic and behavioral factors associated with infestation.

Variable	*n*	% (95% CI)	OR (95% CI)	*p* Value
Age group				
≤10	63/104	60.6 (50.5–69.9)	2.16 (1.00–4.56)	0.013
11–15	185/232	79.7 (73.9–84.6)	2.90 (1.34–3.19)	0.016
16–20	58/117	49.6 (40.3–59.0)	1.97 (0.17–2.28)	0.585
≥21	19/47	40.4 (26.7–55.7)	Ref.	
Sex				
Male	272/429	63.4 (58.6–67.9)	0.52 (0.04–0.72)	0.015
Female	53/71	74.6 (62.7–83.9)	Ref.	
Illiteracy				
Yes	310/440	70.5 (65.9–74.6)	7.15 (3.71–13.95)	<0.001
No	15/60	25.0 (15.1–38.1)	Ref.	
Occupation				
Unemployed	304/445	68.3 (63.7–72.6)	3.83 (1.65–8.89)	<0.001
Farming	12/30	40.0 (23.2–59.2)	1.19 (0.40–3.55)	0.764
Wage earner	9/25	36.0 (18.7–57.4)	Ref.	
Monthly income (NGN)				
≤18,000	312/424	73.6 (69.1–77.7)	7.25 (1.19–88.59)	0.011
>18,000	13/76	17.1 (9.7–27.8)	Ref.	
No. of persons/room/bed				
<4	90/179	50.3 (42.8–57.8)	Ref.	
>4	235/321	73.2 (67.9–79.9)	1.98 (1.08–2.81)	0.004
House structure				
Bricks	100/168	59.5 (51.7–66.9)	Ref.	
Adobe	215/321	66.9 (61.5–72.0)	1.15 (0.18–1.28)	0.071
Palm product	10/11	90.9 (57.1–99.5)	2.20 (1.26–2.61)	0.031
Type of floor				
Sandy	29/31	93.5 (79.2–98.9)	12.17 (2.83–52.34)	0.001
Clay	184/263	70.0 (63.9–75.4)	1.96 (1.34–2.86)	0.001
Cemented	112/283	39.6 (33.9–45.6)	Ref.	
Shared beds and pillows				
Yes	247/352	70.2 (65.0–74.8)	2.11 (1.42–3.14)	<0.001
No	78/148	52.7 (44.4–60.9)	Ref.	
Sharing of clothes				
Yes	105/135	77.8 (69.7–84.3)	2.51 (1.57–3.99)	<0.001
No	220/367	59.9 (54.7–64.9)	Ref.	
Bathing habits				
Regular	193/333	58.0 (52.4–63.3)	0.37 (0.24–0.56)	<0.001
Irregular	132/167	79.0 (72.3–84.5)	Ref.	
Use of bathing soap				
Regular	204/350	58.3 52.9–63.5)	0.36 (0.21–0.53)	<0.001
Irregular	121/150	80.7 (73.2–86.5)	Ref.	

**Table 3:** Clinical features and topographical location of scabies infestation (*n* = 325).

Variable	N (%)
Itching	252 (77.5)
Light	45 (17.9)
Moderate	65 (25.8)
Severe	142 (56.3)
Sleeping disturbance	208 (64.0)
Due to itching	109 (52.4)
Due to pain	32 (15.4)
Others	67 (32.2)
Lymphadenopathy	157 (48.3)
Cervical	35 (22.3)
Axillar	22 (14.0)
Inguinal	100 (64.1)
Infected skin	222 (68.3)
Suppuration	119 (36.6)
No complaints	67 (20.6)
Topographical location of lesions	
Abdomen	115 (35.4)
Inguinal/thigh	62 (19.1)
Wrist	41 (12.6)
Interdigital	46 (14.5)
Legs	23 (7.1)
Elbow	9 (2.8)
Buttock	27 (8.3)
Arms	12 (3.7)
Hands	45 (13.8)
Feet	2 (0.6)
Thorax	1 (0.3)

**Table 4:** Multivariate analysis of factors independently associated with scabies.

Variable	Adjusted Odd Ratio	95% CI	*p* Value
Household income <1 minimum wage	3.23	1.94–3.85	0.026
Sharing of bed and pillow	3.03	2.53–3.21	0.015
Female sex	2.72	1.56–3.52	0.062
Poor housing conditions (no brick house)	2.61	1.94–3.06	<0.001
Unemployment	2.23	1.15–2.59	0.001
Sharing of clothes	2.11	1.88–2.53	0.041
Illiteracy	1.67	1.01–1.93	0.002
Irregular bathing with soap	1.96	0.97–2.13	0.011
Age ≥15 years	0.92	0.42–1.05	0.062
